# A chromosomal reference genome sequence for the malaria mosquito, *Anopheles moucheti*, Evans, 1925

**DOI:** 10.12688/wellcomeopenres.20259.1

**Published:** 2023-11-08

**Authors:** Sandrine N. Nsango, Jean-Pierre Agbor, Diego Ayala, Harriet F. Johnson, Haynes Heaton, Martin G. Wagah, Joanna C. Collins, Ksenia Krasheninnikova, Sarah E. Pelan, Damon-Lee B. Pointon, Ying Sims, James W. Torrance, Alan Tracey, Marcela Uliano Da Silva, Jonathan M. D. Wood, Katharina von Wyschetzki, DNA Pipelines collective, Shane A. McCarthy, Daniel E. Neafsey, Alex Makunin, Mara Lawniczak

**Affiliations:** 1 Faculte de Medecine et des Sciences Pharmaceutiques, Universite de Douala, Douala, Littoral, Cameroon; 2 MIVEGEC, IRD, Montpellier, France; 3 ESV-GAB, Centre Interdisciplinaire de Recherches Médicales de Franceville (CIRMF), Franceville, Gabon; 4 Tree of Life, Wellcome Sanger Institute, Hinxton, England, UK; 5 CSSE, Auburn University, Auburn, Alabama, USA; 6 Department of Genetics, University of Cambridge, Cambridge, England, UK; 7 Department of Immunology and Infectious Diseases, Harvard T.H. Chan School of Public Health, Boston, MA, USA; 8 Infectious Disease and Microbiome Program, Broad Institute, Cambridge, MA, USA

**Keywords:** Anopheles moucheti, African malaria mosquito, genome sequence, chromosomal

## Abstract

We present a genome assembly from an individual male *Anopheles moucheti* (the malaria mosquito; Arthropoda; Insecta; Diptera; Culicidae), from a wild population in Cameroon. The genome sequence is 271 megabases in span. The majority of the assembly is scaffolded into three chromosomal pseudomolecules with the X sex chromosome assembled. The complete mitochondrial genome was also assembled and is 15.5 kilobases in length.

## Species taxonomy

Animalia; Arthropoda; Insecta; Diptera; Culicidae; Anophelinae; Anopheles; *Anopheles moucheti*; Evans, 1925 (NCBI txid:186751).

## Background

*Anopheles moucheti moucheti* (hereafter *An. moucheti*) has the greatest geographical distribution within the *Moucheti* group, which includes *An. bervoetsi* and *An. nigeriensis* ^1, 2^. These species can only be distinguished by slight morphological characters ^3^ and / or using molecular tools ^4, 5^. *An. moucheti* is widely distributed across forested areas of West and Central Africa, and the persistence of this species is linked to its ability to lay eggs in lentic streams and rivers where its larvae develop preferentially and predominate over other malaria vectors ^1, 6^. *An. moucheti* females usually feed indoors and exhibit a preference for feeding on humans ^7^ although the species can also be found in forested areas of Gabon, away from any human presence ^8, 9^. *An. moucheti* is among the most important vectors of human malaria in the equatorial rain forest of Africa ^1, 10^. It is present in a large number of countries, from Guinea to Kenya ^11^, and it has been found naturally infected with *Plasmodium* parasites across Central African countries, sustaining year-round transmission ^1^. For instance, it can show infective biting rates up to 300 bites per person per year in villages located along slow moving rivers ^7, 10^. Moreover, *An. moucheti* has been recently incriminated for contributing to the origin of malaria in humans, being a potential bridge vector for malaria parasites from non-human primates to humans ^8, 9^.

The main genetic work carried out on *An. moucheti* has been focused on differentiation between members of the group. To this end, molecular markers have been developed that are based on the sequence variation in the ribosomal internal transcribed spacer ITS1 region ^5^. Microsatellite loci have also been developed showing a low level of genetic differentiation among populations in Cameroon ^5, 12^. Several polymorphic inversions have been detected in *An. moucheti,* although their role in local adaptation or speciation is not yet understood ^13^. Therefore, genomic characterization of this neglected but major malaria vector, *An. moucheti,* is critical for developing sustainable control strategies for malaria elimination in Central Africa.

The genome of the African malaria mosquito, *Anopheles moucheti*, was sequenced as part of the Anopheles Reference Genomes Project (PRJEB51690). Here we present a chromosomally complete genome sequence for *Anopheles moucheti*, based on a single male specimen from Ebogo, Cameroon.

## Genome sequence report

The genome was sequenced from a single male *Anopheles moucheti* collected from Ebogo, Cameroon (3.384, 11.466). A total of 41-fold coverage in Pacific Biosciences single-molecule HiFi long reads (N50 13.064 kb) were generated. Primary assembly contigs were scaffolded with chromosome conformation Hi-C data from a sibling male mosquito. Manual assembly curation corrected 48 missing joins or misjoins and removed 3 haplotypic duplications, reducing the scaffold number by 7.8% and reducing assembly size by 1.8%.

**Figure 1.**
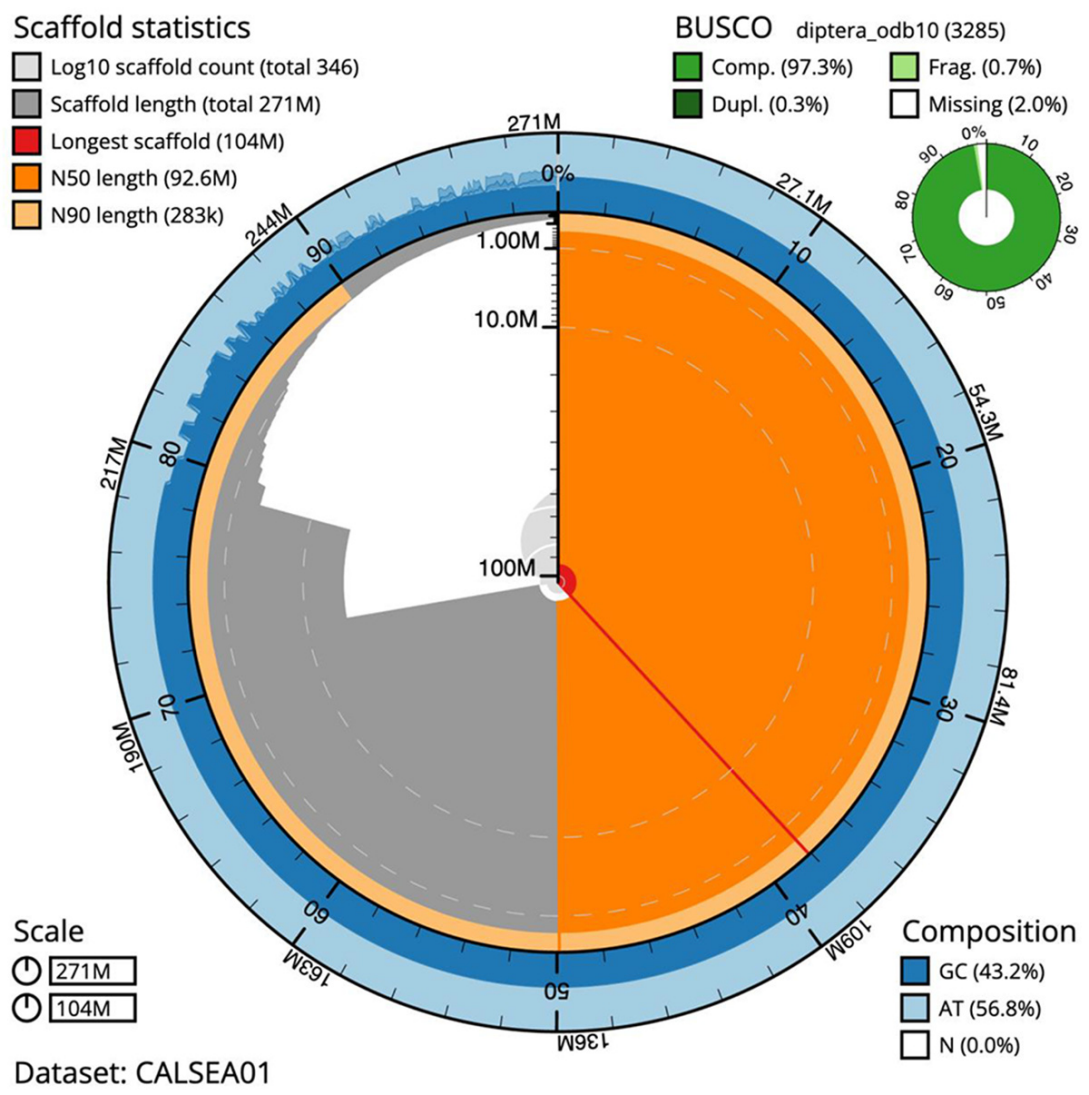
Snail plot summary of assembly statistics for *Anopheles moucheti* assembly idAnoMoucSN_F20_07. The main plot is divided into 1,000 size-ordered bins around the circumference with each bin representing 0.1% of the 271,332,225 bp assembly. The distribution of sequence lengths is shown in dark grey with the plot radius scaled to the longest sequence present in the assembly (103,511,506 bp, shown in red). Orange and pale-orange arcs show the N50 and N90 sequence lengths (92,649,126 and 283,162 bp), respectively. The pale grey spiral shows the cumulative sequence count on a log scale with white scale lines showing successive orders of magnitude. The blue and pale-blue area around the outside of the plot shows the distribution of GC, AT and N percentages in the same bins as the inner plot. A summary of complete, fragmented, duplicated and missing BUSCO genes in the diptera_odb10 set is shown in the top right. An interactive version of this figure is available at https://blobtoolkit.genomehubs.org/view/idAnoMoucSN_F20_07/dataset/CALSEA01/snail.

The final assembly has a total length of 271 Mb in 345 sequence scaffolds with a scaffold N50 of 92.649 Mb ( Table 1). 84.7% of the assembly sequence was assigned to three chromosomal-level scaffolds, representing two autosomes (numbered and oriented against the *An. gambiae* PEST strain assembly AgamP3 ( 14; GCF_000005575.2) with chromosome arms homologies for *Myzomyia* series taken from 15), and the X sex chromosome ( Figure 1– Figure 6; Table 2).

**Table 1.** Genome data for *Anopheles moucheti*, idAnoMoucSN_F20_07.

*Project accession data*	
Assembly identifier	idAnoMoucSN_F20_07
Species	*Anopheles moucheti*
Specimen	idAnoMoucSN-F20_07
NCBI taxonomy ID	186751
BioProject	PRJEB53257
BioSample ID	ERS10527373
Isolate information	male, whole organism
*Raw data accessions*	
PacificBiosciences SEQUEL II	ERR9439507
Hi-C Illumina	ERR9356830
PolyA RNA-Seq Illumina	ERR9356831, ERR9356832
*Genome assembly*	
Assembly accession	GCA_943734755
*Accession of alternate haplotype*	GCA_943734785
Span (Mb)	271.317
Number of contigs	406
Contig N50 length (Mb)	4.744
Number of scaffolds	345
Scaffold N50 length (Mb)	92.649
Longest scaffold (Mb)	103.512
BUSCO * genome score	C:97.6%[S:97.3%,D:0.3%], F:0.7%,M:2.0%,n:3285

* BUSCO scores based on the diptera_odb10 BUSCO set using BUSCO 5.3.2. C=complete [S=single copy, D=duplicated], F=fragmented, M=missing, n=number of orthologues in comparison. A full set of BUSCO scores is available at https://blobtoolkit.genomehubs.org/view/idAnoMoucSN_F20_07/dataset/CALSEA01/busco.

**Figure 2.**
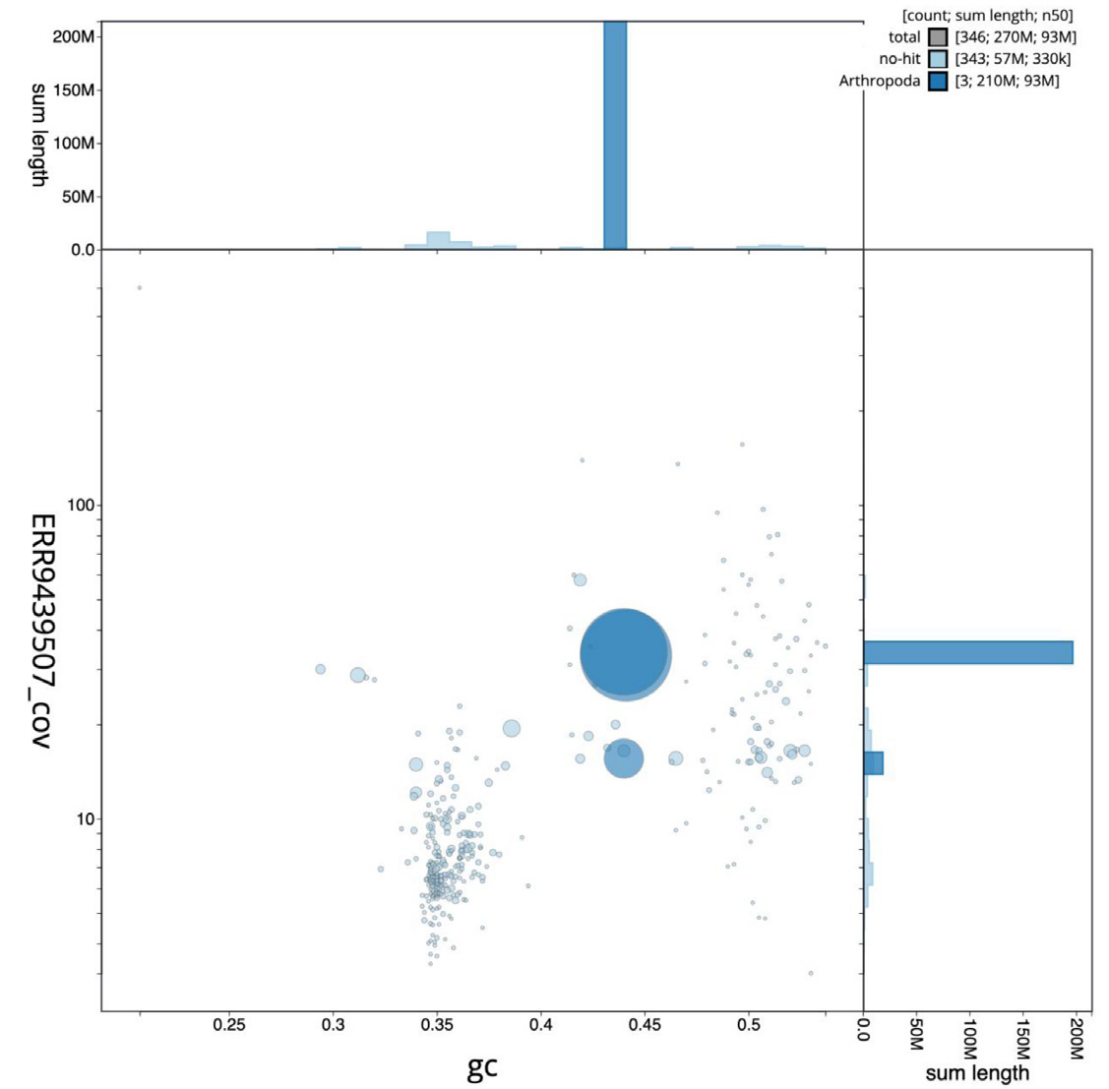
Blob plot of base coverage in idAnoMoucSN-F20_07 PacBio HiFi reads against GC proportion for *An. moucheti* assembly idAnoMoucSN_F20_07. Chromosomes are coloured by phylum. Circles are sized in proportion to chromosome length. Histograms show the distribution of chromosome length sum along each axis. An interactive version of this figure is available at https://blobtoolkit.genomehubs.org/view/idAnoMoucSN_F20_07/dataset/CALSEA01/blob.

**Figure 3.**
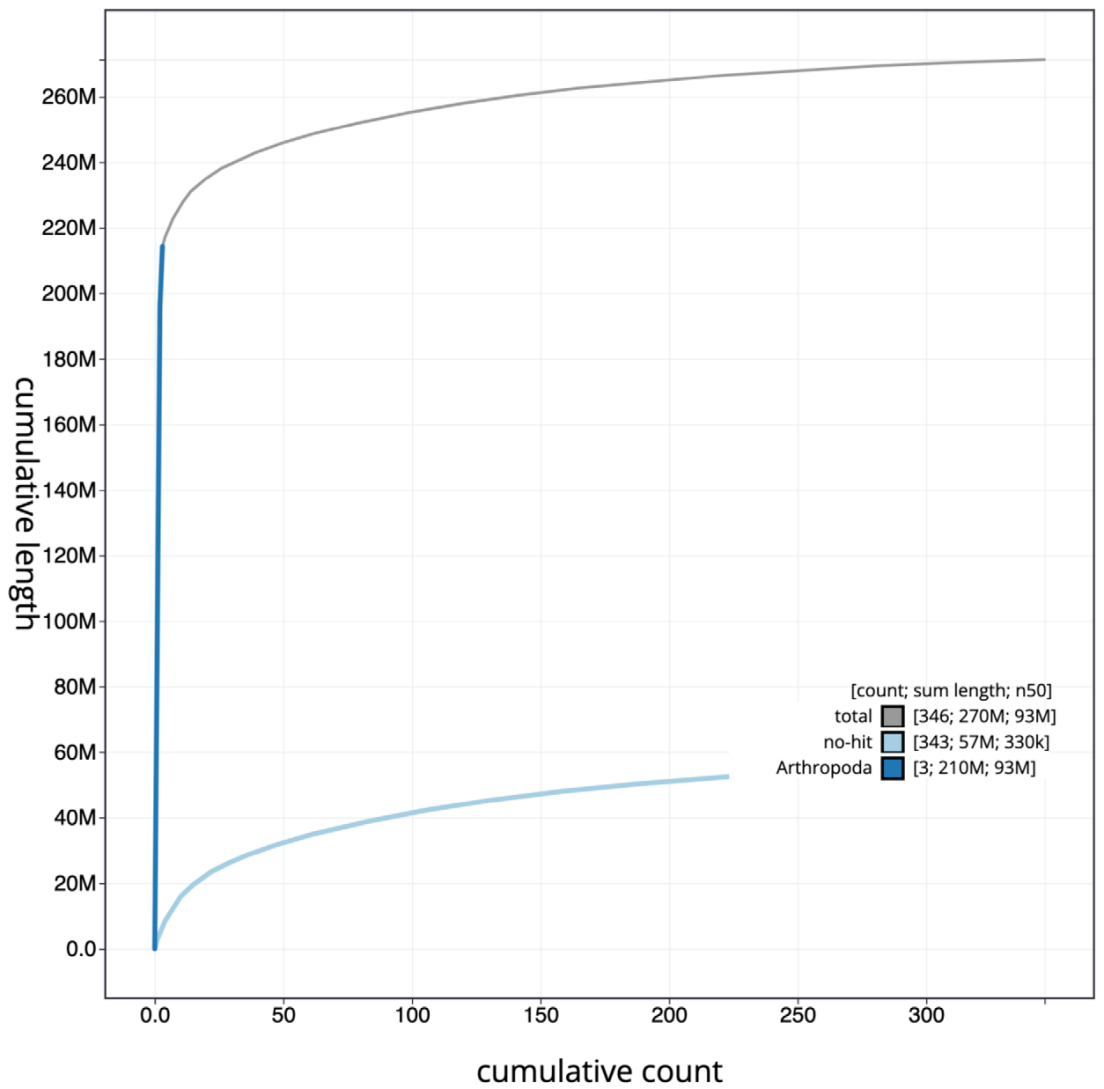
Cumulative chromosome length for *An. moucheti* assembly idAnoMoucSN_F20_07. The grey line shows cumulative length for all chromosomes. Coloured lines show cumulative lengths of chromosomes assigned to each phylum using the buscogenes taxrule. The interactive version of this figure is available at https://blobtoolkit.genomehubs.org/view/idAnoMoucSN_F20_07/dataset/CALSEA01/cumulative.

**Figure 4.**
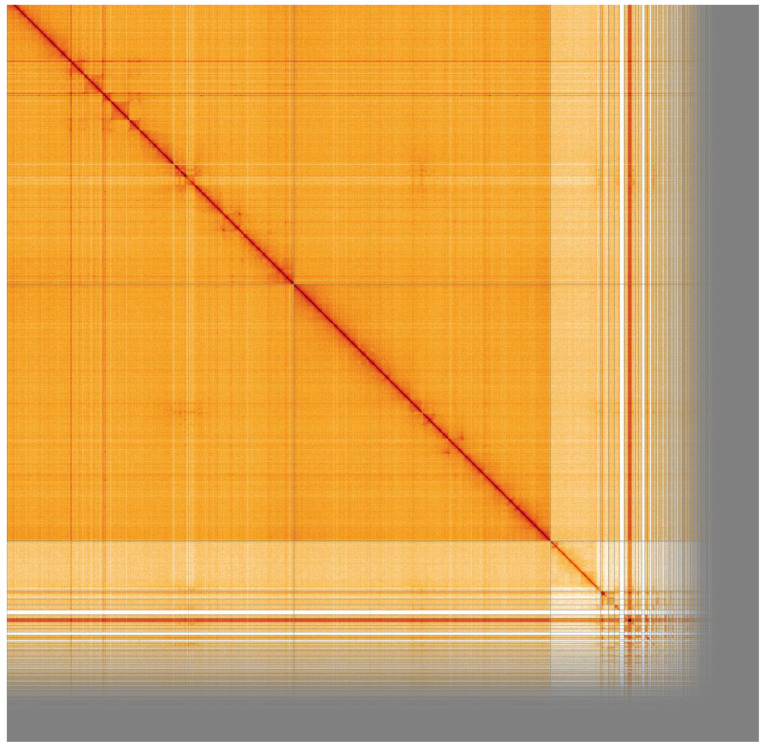
Genome assembly of *An. moucheti*, idAnoMoucSN_F20_07: Hi-C contact map. Visualised in HiGlass. Chromosomes are arranged in size order from left to right and top to bottom. X chromosome signal is lower as individuals used for both PacBio and HiC are males. The interactive Hi-C map can be viewed at https://genome-note-higlass.tol.sanger.ac.uk/l/?d=GZ6DShODRcSMNgw7a0UIfQ.

**Figure 5.**
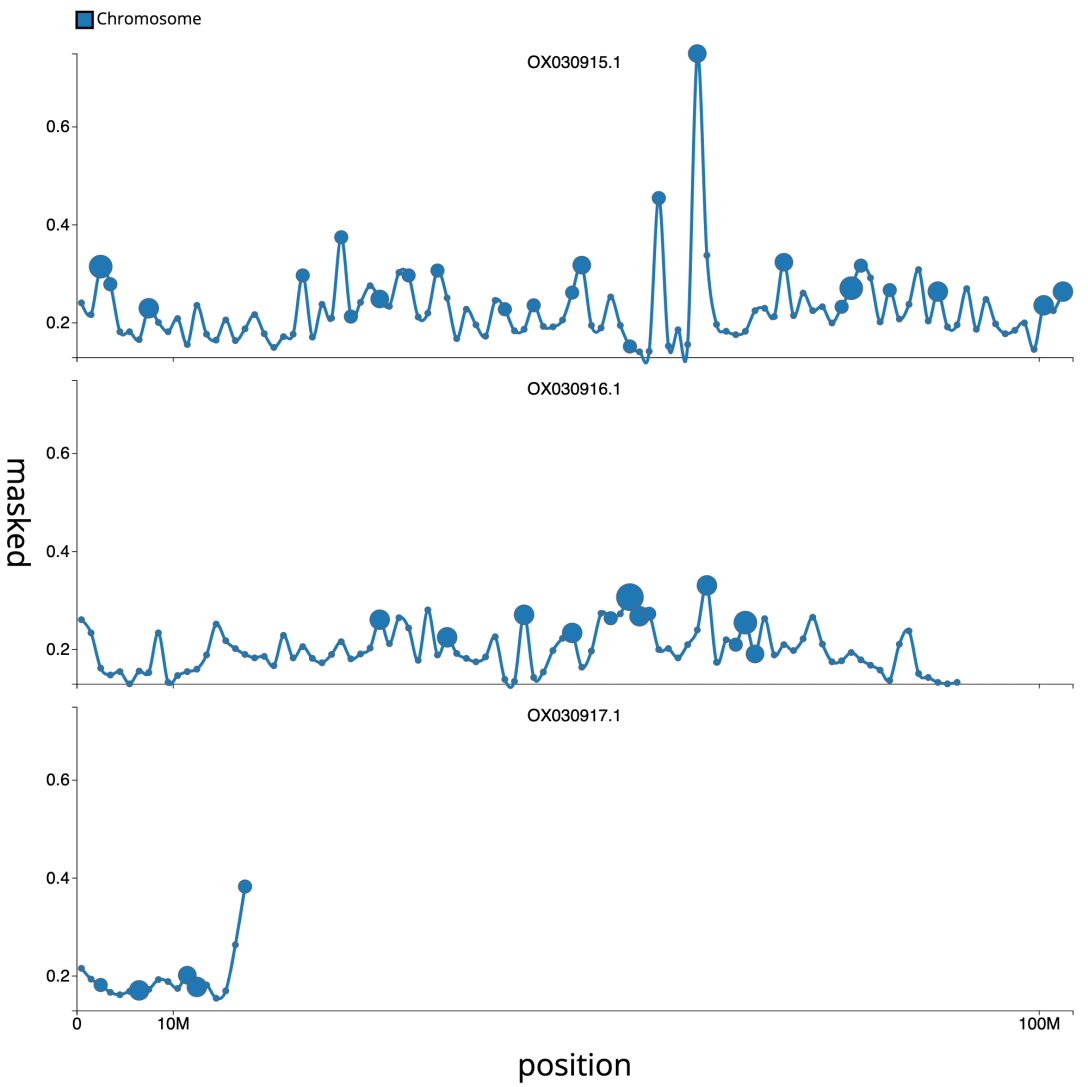
Distribution plot of repeat masked sequence proportion against genomic position for chromosomes of *An. moucheti*, idAnoMoucSN_F20_07. 1Mb windows with assembly gaps highlighted by larger blue circles, size is proportional to total size of gaps in the window (max 7800bp per window).

**Figure 6.**
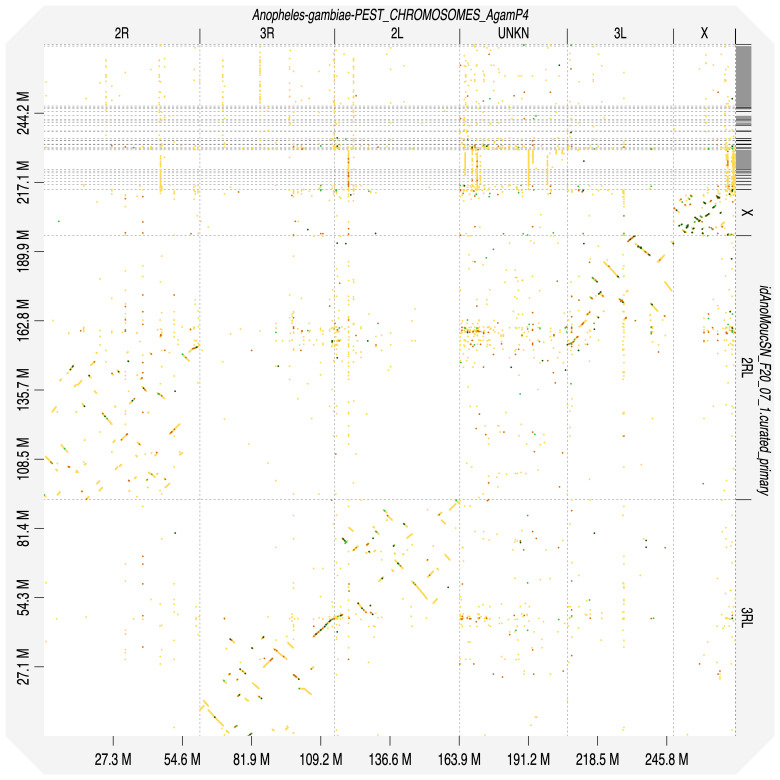
Alignment dotplot between genome assemblies of *An. moucheti* (idAnoMoucSN_F20_07) and *An. gambiae*, AgamP4 (PEST). Visualised in DGenies. Chromosome arms correspondence (moucheti-gambiae): 2R-2R, 2L-3L, 3R-3R, 3L-2L in agreement with 15.

**Table 2.** Chromosomal pseudomolecules in the genome assembly of *An. moucheti*, idAnoMoucSN_F20_07.

INSDC accession	Chromosome	Size (Mb)	Count	Gaps
OX030915.1	2RL	103.512	1	30
OX030916.1	3RL	92.649	1	15
OX030917.1	X	18.119	1	6
OX030918.1	MT	0.016	1	0
	X Unlocalised	15.547	98	0
	Unplaced	41.489	244	10

The assembly has a BUSCO 5.3.2 ^16^ completeness of 97.6% using the diptera_odb10 reference set. While not fully phased, the assembly deposited is of one haplotype. Contigs corresponding to the second haplotype have also been deposited.

## Methods

### Sample acquisition and nucleic acid extraction

*Anopheles moucheti* offspring were reared from a wild caught gravid female collected from Ebogo, Cameroon (3.384, 11.466) by Sandrine Eveline Nsango. A single male idAnoMoucSN-F20_07 was used for Pacific BioSciences, a single sibling male idAnoMoucSN-F20_09 was used for Arima Hi-C.

For high molecular weight (HMW) DNA extraction one whole insect (idAnoMoucSN-F20_07) was disrupted by manual grinding with a blue plastic pestle in Qiagen MagAttract lysis buffer ^17^ and then extracted using the Qiagen MagAttract HMW DNA extraction kit with two minor modifications including halving volumes recommended by the manufacturer due to small sample size ( *Anopheles* mosquitoes typically weigh 2–3 mg) and running two elution steps of 100 μl each to increase DNA yield. The quality of the DNA was evaluated using an Agilent FemtoPulse to ensure that most DNA molecules were larger than 30 kb, and preferably > 100 kb. In general, single mosquito extractions ranged in total estimated DNA yield from 200 ng to 800 ng, with an average yield of 500 ng. Low molecular weight DNA was removed using 0.8X AMpure XP purification. DNA was sheared to an average fragment size of 12–20 kb using a Diagenode Megaruptor 3 at speeds ranging from 27 to 30. Sheared DNA was purified using AMPure PB beads with a 1.8X ratio of beads to sample to remove the shorter fragments and concentrate the DNA sample. The concentration and quality of the sheared and purified DNA was assessed using a Nanodrop spectrophotometer and Qubit Fluorometer with the Qubit dsDNA High Sensitivity Assay kit. Fragment size distribution was evaluated by running the sheared and cleaned sample on the FemtoPulse system once more. The median DNA fragment size for *Anopheles* mosquitoes was 15 kb and the median yield of sheared DNA was 200 ng, with samples typically losing about 50% of the original estimated DNA quantity through the process of shearing and purification.

For Hi-C data generation, a sibling male mosquito specimen (idAnoMoucSN-F20_09) was used as input material for the Arima V2 Kit according to the manufacturer’s instructions for animal tissue. This approach of using a sibling was followed in order to enable all material from a single specimen to contribute to the PacBio data generation given we were not always able to meet the minimum suggested guidance of starting with > 300 ng of HMW DNA from a specimen. Samples proceeded to the Illumina library prep stage even if they were suboptimal (too little tissue) going into the Arima reaction.

To assist with annotation, which will be made available through VectorBase ^18^ in due course, RNA was extracted from separate whole unrelated lab-reared male and wild-caught female insect specimens (idAnoMoucSN-F4_01, idAnoMoucSN-F7_x) using TRIzol, according to the manufacturer’s instructions. RNA was then eluted in 50 μl RNAse-free water, and its concentration was assessed using a Nanodrop spectrophotometer and Qubit Fluorometer using the Qubit RNA Broad-Range (BR) Assay kit. Analysis of the integrity of the RNA was done using Agilent RNA 6000 Pico Kit and Eukaryotic Total RNA assay. Samples were not always ideally preserved for RNA, so qualities varied but all were sequenced anyway.

### Sequencing

We prepared libraries as per the PacBio procedure and checklist for SMRTbell Libraries using Express TPK 2.0 with low DNA input. Every library was barcoded to support multiplexing. Final library yields ranged from 20 ng to 100 ng, representing only about 25% of the input sheared DNA. Libraries from two specimens were typically multiplexed on a single 8M SMRT Cell. Sequencing complexes were made using Sequencing Primer v4 and DNA Polymerase v2.0. Sequencing was carried out on the Sequel II system with 24-hour run time and 2-hour pre-extension. For Hi-C data generation, following the Arima Hi-C V2 reaction, samples were processed through Library Preparation using a NEB Next Ultra II DNA Library Prep Kit and sequenced aiming for 100x depth. RNA libraries were created using the directional NEB Ultra II stranded kit. Sequencing was performed by the Scientific Operations core at the Wellcome Sanger Institute on Pacific Biosciences SEQUEL II (HiFi), Illumina NovaSeq 6000 (10X and Hi-C), or Illumina HiSeq 4000 (RNAseq) instruments.

### Genome assembly

Assembly was carried out with Hifiasm ^19^; haplotypic duplications were identified and removed with purge_dups ^20^. The assembly was then scaffolded with Hi-C data ^21^ using SALSA2 ^22^. The assembly was checked for contamination as described previously ^23^. Manual curation was performed using gEVAL ^24^, HiGlass ^25^ and Pretext ^26^. The mitochondrial genome was assembled using MitoHiFi ^27^, which performs annotation using MitoFinder ^28^. The genome was analysed and BUSCO scores were generated within the BlobToolKit environment ^29^. Synteny analysis was performed with D-GENIES ^30^ and minimap2 ^31^. Table 3 contains a list of all software tool versions used, where appropriate.

**Table 3.** Software tools used.

Software tool	Version	Source
hifiasm	0.14	19
purge_dups	1.2.3	20
SALSA2	2.2-4c80ac1	22
MitoHiFi	2	27
gEVAL	N/A	24
HiGlass	1.11.6	25
PretextView	0.1.x	26
BlobToolKit	3.4.0	32
BUSCO	5.3.2	16
D-GENIES	1.4	30
minimap2	2.24	31

## Ethics/compliance issues

The genetic resources accessed and utilised under this project were done so in accordance with the UK ABS legislation (Nagoya Protocol (Compliance) (Amendment) (EU Exit) Regulations 2018 (SI 2018/1393)) and the national ABS legislation within the country of origin, where applicable.

## Data Availability

European Nucleotide Archive: *Anopheles moucheti* genome assembly, idAnoMoucSN_F20_07. Accession number PRJEB53257; https://identifiers.org/bioproject/PRJEB53257. The genome sequence is released openly for reuse. The *Anopheles moucheti* genome sequencing initiative is part of the Anopheles Reference Genomes project PRJEB51690. All raw sequence data and the assembly have been deposited in INSDC databases. Raw data and assembly accession identifiers are reported in Table 1.

## Author information

Members of the Sanger Scientific Operations: DNA Pipelines collective are listed here: https://doi.org/10.5281/zenodo.4790455.
