# Supplementary material for: A chromosomal reference genome sequence for the malaria mosquito, Anopheles moucheti, Evans, 1925
Source: Wellcome Open Res. 2023 Nov 8;8:507. [Version 1] doi: 10.12688/wellcomeopenres.20259.1 (PMC10690039; doi:10.12688/wellcomeopenres.20259.1)
Supplement: Peer review history

# Reviewer report: Reviewer response for version 1

**DOI:** 10.21956/wellcomeopenres.22437.r69865

**Published:** 2023-11-30

**Authors:** Mary Ann McDowell, Michelle Huang

**Affiliations:** 1 University Notre Dame, Notre Dame, Indiana, USA; 2 Biological Sciences, University of Notre Dame, Notre Dame, Indiana, USA

This data note by Nsango *et al.* present the methods and tools used for generating the first *Anopheles moucheti* genome assembly. Though *An. moucheti* is an important vector for human malaria, preferentially feeding indoor and on humans, and potentially acting as a bridge vector from non-human primates to humans, the authors explain genomic characterization of the species remains limited. Here, they used an individual male *An. moucheti* reared from a wild-caught gravid female for PacBio sequencing and a single sibling male for Arima Hi-C to construct three chromosomal pseudomolecules, the X sex chromosome, and the mitochondrial genome. They describe their methods from sample acquisition through genome assembly in appropriate detail to be replicated by others.

 In Figure 3, the figure caption may be more descriptive if labeled “cumulative scaffold length” instead of “cumulative chromosome length” as only the dark blue “Arthropoda” line represents the chromosomes. Additionally, the figure legend truncates the “no-hit” line at around 225, which can be confusing as it is expected to end around 340. Moving the figure legend would be ideal. If this is not possible using BlobToolKit, setting the curve origin to “X” could be an improvement, as the “Arthropoda” line will originate where the “no-hit” line ends.

Are sufficient details of methods and materials provided to allow replication by others?

Yes

Is the rationale for creating the dataset(s) clearly described?

Yes

Are the datasets clearly presented in a useable and accessible format?

Yes

Are the protocols appropriate and is the work technically sound?

Yes

Reviewer Expertise:

Vector Biology, Genomics

We confirm that we have read this submission and believe that we have an appropriate level of expertise to confirm that it is of an acceptable scientific standard.

# Reviewer report: Reviewer response for version 1

**DOI:** 10.21956/wellcomeopenres.22437.r69866

**Published:** 2023-11-16

**Authors:** Igor V Sharakhov

**Affiliations:** 1 Department of Entomology, Virginia Polytechnic Institute and State University, Blacksburg, USA

The article by Nsango and co-authors present a new genome assembly obtained from an individual male *Anopheles moucheti*. The genome was sequenced using the PacBio HiFi procedure and scaffolded into three chromosomal pseudomolecules using Hi-C data obtained from a single sibling mosquito male. The complete mitochondrial genome was also assembled. The rationale for creating the *Anopheles moucheti* genome is clearly described as the species is an antropophilic malaria vector and a potential bridge vector for malaria parasites from non-human primates to humans. The manuscript provides sufficient details of methods and materials to allow replication by others. 

 It would be useful if chromosomes or chromosomal arms (if possible) are labeled in Figure 4. Have centromeric regions been captured either by reads or contigs? 

 There are high frequency interaction spots outside the main diagonal in Figure 4. These could be indicative of polymorphic inversions. If indeed, this study assembled both haplotypes, then it is possible to test if they differ in the structural arrangements. This can be the case if the sequenced individual was a heterozygote. Also, building a Hi-C contact map for the second haply could answer this question.

Are sufficient details of methods and materials provided to allow replication by others?

Yes

Is the rationale for creating the dataset(s) clearly described?

Yes

Are the datasets clearly presented in a useable and accessible format?

Partly

Are the protocols appropriate and is the work technically sound?

Yes

Reviewer Expertise:

Mosquito genetics, cytogenetics, genomics

I confirm that I have read this submission and believe that I have an appropriate level of expertise to confirm that it is of an acceptable scientific standard.
